# Disaggregating Census Data for Population Mapping Using Random Forests with Remotely-Sensed and Ancillary Data

**DOI:** 10.1371/journal.pone.0107042

**Published:** 2015-02-17

**Authors:** Forrest R. Stevens, Andrea E. Gaughan, Catherine Linard, Andrew J. Tatem

**Affiliations:** 1 Department of Geography and Geosciences, University of Louisville, Louisville, Kentucky, United States of America; 2 Fonds National de la Recherche Scientifique (F.R.S.-FNRS), Rue d’Egmont 5, B-1000 Brussels, Belgium; 3 Biological Control and Spatial Ecology, Université Libre de Bruxelles, CP 160/12, Avenue FD Roosevelt 50, B-1050 Brussels, Belgium; 4 Department of Geography and Environment, University of Southampton, Highfield, Southampton SO17 1BJ, United Kingdom; 5 Fogarty International Center, National Institutes of Health, Bethesda, MD 20892, United States of America; Northwestern University, UNITED STATES

## Abstract

High resolution, contemporary data on human population distributions are vital for measuring impacts of population growth, monitoring human-environment interactions and for planning and policy development. Many methods are used to disaggregate census data and predict population densities for finer scale, gridded population data sets. We present a new semi-automated dasymetric modeling approach that incorporates detailed census and ancillary data in a flexible, “Random Forest” estimation technique. We outline the combination of widely available, remotely-sensed and geospatial data that contribute to the modeled dasymetric weights and then use the Random Forest model to generate a gridded prediction of population density at ~100 m spatial resolution. This prediction layer is then used as the weighting surface to perform dasymetric redistribution of the census counts at a country level. As a case study we compare the new algorithm and its products for three countries (Vietnam, Cambodia, and Kenya) with other common gridded population data production methodologies. We discuss the advantages of the new method and increases over the accuracy and flexibility of those previous approaches. Finally, we outline how this algorithm will be extended to provide freely-available gridded population data sets for Africa, Asia and Latin America.

## Introduction

Accurate spatial data sets that represent the distributions of human populations are critical in many health, economic, and environmental fields across various temporal and spatial scales [1–3]. Considering an estimated world population increase of 2.3 billion people between 2011 and 2050, with more than 50% of that growth absorbed into urban areas [4], the ramifications of having accurate population information has never been more important. Demand is increasing for more contemporary, easily-updatable population data as research and decision-making become more complex and operates at sub census-unit scales [5, 6]. To satisfy this demand researchers are increasingly turning to remotely sensed data and other geospatial data sets to refine the process of producing high resolution estimates of population density. But, to make the most of these data sources, new methodologies are needed to more accurately estimate human population distributions.

Gridded population data sets can vary substantially in their depiction of population distributions, especially for resource poor countries where recent and detailed census and country-specific geospatial data are often limited [7]. Previous efforts to generate gridded population data sets have utilized areal interpolation techniques, which include basic dasymetric approaches [8] often in conjunction with ancillary data [1] or, alternatively, statistical modeling methods [9]. Widely-used global data sets that rely on an areal weighting scheme include the freely available Gridded Population of the World (GPW) database, versions 2 and 3, and the Global Rural Urban Mapping Project (GRUMP) [8, 10]. GRUMP differs from GPW by incorporating urban-rural designations in the spatial reallocation of population for each census unit [11], primarily derived from satellite nightlights, while GPW is a simple redistribution across census unit. Other large-area population data sets rely on ancillary data to spatially weight population density within a given administrative unit. Such data sets include the LandScan Global Population database [12, 13], the gridded data sets produced by the United Nations Environment Programme (UNEP) for Latin America, Africa, and Asia [14–16], and the AfriPop and AsiaPop projects which provide freely-available gridded population data for Africa and Asia [5, 17–19].

Each of these data products differ in their modeling approach and transparency, the input data and reliability of those data sources, and how input variables interact with each other to determine population distribution. Remotely sensed information has been applied for decades either as the main source for human population estimation [20–22] or alternatively used as a supplementary data source for use in spatially refining census population estimates [17, 23–25]. The use of remotely sensed data is especially important for mapping human population in countries that do not have reliable census data collection [5]. Recent studies focused on mapping populations in Haiti in 2003, Pakistan in 1998 and Western Kenya in 1999 take advantage of the increased availability of multi-scale remotely-sensed and geospatial data [23, 26, 27]. These studies use semi-automated classification algorithms combined with a dasymetric mapping approach for generating fine-scale population data sets. While such studies provide important contributions towards the advancement of accurate gridded population mapping, they can be difficult to apply across regional to continental spatial scales due to a reliance on high resolution imagery that is often expensive to obtain and challenging to process.

Here we propose an approach that complements such methods, with flexibility that allows for incorporating global, large scale data sets of both continuous and discrete covariates when finer scale data do not exist or are difficult to process over large areas. We describe a dasymetric redistribution approach, using population counts from census data and a weighting scheme which is based on a “Random Forest,” nonparametric predictive model [28, 29]. The flexibility of this modeling framework allows for the incorporation of remotely sensed and geospatial data from multiple scales into the weighting portion of the dasymetric model. The approach benefits from non-parametric statistical predictions based on the best ancillary data available, but also anchors those predictions across space to the best available, contemporary administrative boundary-linked GIS census data.

We present the methodology using case studies of Kenya (KEN), Vietnam (VNM), and Cambodia (KHM). These three countries represent a cross-section of census spatial scales and also reflect the range in ancillary data available for use in the algorithm. We present a comparison of the population maps and their accuracy for each country with output from the simpler, widely used methods used to create the Afri/AsiaPop, GRUMP and GPW datasets.

## Materials and Methods

### Data Description

Country-specific census data were collected from the National Institute of Statistics for Cambodia, the National Statistics Office in Vietnam, and the National Bureaus of Statistics for Kenya (Table 1). These data were matched to GIS-delineated administrative boundaries for the village (Cambodia), Tinh (Vietnam) and Sublocation (Kenya) levels. These administrative levels provided the finest level administrative unit available at the time of analysis. For use in the Random Forest model, the log population density is used as a response variable and this process is explained in more detail in Section 2.4 with an expanded discussion on the choice of performing a log transformation provided in Section 4.3.

**Table 1:** Country-specific data sources and variable names used for population density estimation used for dasymetric weights.

Type	Variable Name(s)*	Description	Cambodia Data	Vietnam Data	Kenya Data
Census		Country-specific census and scale	2008, Admin-level 3	1999, Admin-level 4	1999, Admin-level 5
Land Cover	lan_cls011, lan_dst011	Cultivated terrestrial lands	MDA Landcover, 30m	MDA Landcover, 30m	GlobCover, 300m
	lan_cls040, lan_dst040	Woody / Trees			
	lan_cls130, lan_dst130	Shrubs			
	lan_cls140, lan_dst140	Herbaceous			
	lan_cls150, lan_dst150	Other terrestrial vegetation			
	lan_cls160, lan_dst160	Aquatic vegetation			
	lan_cls190, lan_dst190	Urban area			
	lan_cls200, lan_dst200	Bare areas			
	lan_cls210, lan_dst210	Water bodies			
	lan_cls230, lan_dst230	No data, cloud/shadow			
	lan_cls240, lan_dst240	Rural settlement			
	lan_cls250, lan_dst250	Industrial area			
	lan_clsBLT, lan_dstBLT	Built, merged urban/rural class			
Continuous					
Raster-Format	Npp	Annual NPP, 2010	MODIS 17A3	MODIS 17A3	MODIS 17A3
	Lig	Lights at night	Suomi VIIRS-Derived	Suomi VIIRS-Derived	Suomi VIIRS-Derived
	Tem	Mean temperature, 1950–2000	WorldClim/BioClim	WorldClim/BioClim	WorldClim/BioClim
	Pre	Mean precipitation, 1950–2000	WorldClim/BioClim	WorldClim/BioClim	WorldClim/BioClim
	Ele	Elevation	HydroSHEDS [31]	HydroSHEDS[31]	HydroSHEDS[31]
	ele_slope	Slope	HydroSHEDS-Derived	HydroSHEDS-Derived	HydroSHEDS-Derived
Converted					
Vector-Format	roa_dst	Distance to roads	World Food Programme	OSM [39]	Kenyan Bureau of Stat.
	riv_dst	Distance to rivers/streams	World Food Programme	VMAP0 *hydro/watrcrsl*	VMAP0 *hydro/watrcrsl*
	pop_cls, pop_dst	Generic populated places	VMAP0 *merged* ^†^	VMAP0 *merged* ^†^	Tatem, et al. [46]
	wat_cls, wat_dst	Water bodies	World Food Programme	OSM [39]	VMAP0 *hydro/inwatera*
	pro_cls, pro_dst	Protected areas	WDPA, IUCN [47]	WDPA, IUCN [47]	WDPA, IUCN [47]
	can_cls, can_dst	Canals	World Food Programme		
	com_cls, com_dst	Communities	World Food Programme		
	dis_cls, dis_dst	District seats	World Food Programme		
	cit_cls, cit_dst	Cities		OSM [39]	
	ham_cls, ham_dst	Hamlets		OSM [39]	
	vil_cls, vil_dst	Villages	KHM Census 2008	OSM [39]	
	sub_cls, sub_dst	Suburbs		OSM [39]	
	tow_cls, tow_dst	Towns		OSM [39]	
	poi_cls, poi_dst	Populated Points		OSM [39]	
	rai_cls, rai_dst	Railways		OSM [39]	
	fac_cls, fac_dst	Generic health facilities	HR-COD [48]		
	cli_cls, cli_dst	Health clinics			Noor, et al. [44]
	dsp_cls, dsp_dst	Dispensaries	HR-COD [48]		Noor, et al. [44]
	hos_cls, hos_dst	Hospitals			Noor, et al. [44]
	sch_cls, sch_dst	Schools	HR-COD [48]		Kenya Open Data [49]
	set_cls, set_dst	Settlement points	World Food Programme		
	bui_cls, bui_dst	Built land cover			Tatem, et al. [46]

* The variable names are used in Random Forest model output and throughout the text as reference to the specific data they were derived from. The first three letters are derived from the data type (e.g. “*lan*” indicates land cover) and the last three letters, if present, indicates what type of data each variable represents (e.g. “_*cls*” is a binary classification and “_*dst*” is a calculated Euclidean distance-to variable.

^†^ The default data for populated places is merged from several VMAP0 data sources. There are three VMAP0 data sets used: The point data *pop/builtupp* and *pop/mispopp* are buffered to 100 m and merged with the *pop/builtupa* polygons creating avector-based built layer. This layer is then converted to binary class and distance-to rasters for use in modeling.

Population distribution is often highly correlated with land cover types and we incorporate land cover information using one of two thematic land cover classification data sets. For Cambodia and Vietnam, we use EarthSat GeoCover Land Cover Thematic Mapper (TM) data from MDA Federal [30] (Table 1). The GeoCover dataset provides consistent global mapping of 13 land cover classes at a 30-meter spatial resolution and derived from circa 2005 imagery [30]. GlobCover data, which are derived from the ENVISAT satellite mission's MERIS (Medium Resolution Image Spectrometer) imagery, were used for Kenya (Table 1). GeoCover imagery classes were re-coded to be consistent with those land cover classes used by GlobCover and the aggregated classes used in the AsiaPop [19] and AfriPop [25] methodologies. GeoCover data (30 m) were majority aggregated (scaled-up) and GlobCover data (300 m) were resampled (scaled-down) by nearest neighbor to a square pixel resolution of 8.33 x 10^–4^ degrees (approximately 100 meters at the equator).

Land cover data are complemented by digital elevation data and its derived slope estimates, primarily from the SRTM-based HydroSheds data [31]. We also include MODIS-derived, MOD17A3 estimates of net primary productivity (NPP) [32] as well as observed lights at night, mosaicked from Suomi National Polar-orbiting Partnership (NPP) Visible Infrared Imaging Radiometer Suite (VIIRS) data, standardized and provided as a global coverage [33]. Within-country climatic spatial variation is also incorporated, by using WorldClim/BioClim 1950–2000 mean annual precipitation (BIO12) and mean annual temperature (BIO1) estimates [34].

In addition to land cover and associated raster data sets, we also include geospatial data that may correlate with human population presence on the landscape such as networks of roads and waterways; large water bodies; settlement or populated place locations; protected area delineations; and various “facility” locations (e.g. health clinics, hospitals, schools). The specific data sets available vary widely from country to country, but in almost all cases the most comprehensive, contemporary datasets that are freely available were used. In the absence of country-specific data for many of these features we extract National Geospatial-Intelligence Agency (NGA) Vector Map Level 0 (VMAP0) data [35]. In many cases the VMAP0 data are the most coarse and non-contemporaneous available, however their world-wide coverage and consistency in level of processing make them a useful base data set, especially in the absence of alternative data for roads, rivers, water bodies and built-up areas. The data used in the case studies presented here are summarized in Table 1.

### Data Preparation

**Fig 1:**
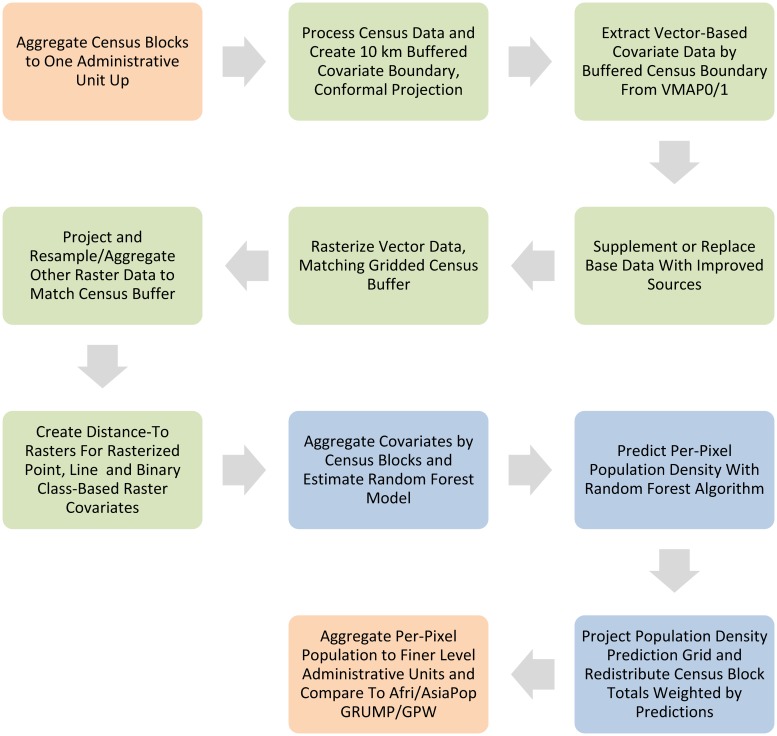
This figure represents the general structure of the data processing and map production procedure used to compare the methodology outlined in this paper to the AfriPop/AsiaPop, GRUMP, and GPW methodologies. The orange boxes represent items that are specific to the research presented here and not part of end-user map data product generation. The green boxes represent data pre-processing stages. Items in blue represent Random Forest model estimation, per-pixel prediction and dasymetric redistribution of census counts.

The general process used for the data preparation, modeling and validation is outlined in Fig. 1. The steps in green represent the following data preparation tasks, all performed using a standalone Python programming language (version 2.6, www.python.org) script that uses the ArcGIS 10.0 SP1 [36] *arcpy* module and associated extensions for analysis. Slight differences exist in the processing and the data sources used for each country, but these are thoroughly documented in the metadata that accompanies each set of final population maps (see attached S1 File for an example).

Before processing the covariates, the administrative boundary-linked census population count data are converted to raster form by projecting the data into a conformal projection most appropriate for each country (e.g. UTM). Projection is necessary for calculating the distance-to covariates included in the model. The projected census data boundaries, which usually correspond to the national borders of each country, are then buffered by 10 km. This buffered polygon is used in order to minimize edge effects associated with near-border populated areas. The buffered polygon is then converted to a raster grid, with approximately 100 meter by 100 meter pixels, and serves as a template for all other covariates to be projected and subset to for each country.

The vector and raster class-based data, (e.g. individual land cover classes, water bodies, protected area presence/absence, etc.) are first projected and subset to match the buffered national borders. These class data are then converted to binary masks, creating a binary covariate (i.e. “_cls” variables in Table 1). From these masks a distance-to-class raster is calculated for each dataset (i.e. “_dst” variables in Table 1). The one special case for class-based data occurs in the treatment of the “Built” land cover class (“lan_BLT_” in Table 1). Using the same methodology outlined for both the AfriPop and AsiaPop projects [17, 19], but using the MODIS 500 m Global Urban Extent product (2001–2002) [37, 38], the “Built” class in each land cover dataset is categorized as either “Rural” or “Urban” based on whether the pixel location is within a rural/urban extent. Furthermore, the built areas may be adjusted on a pixel-by-pixel basis using other ancillary data sources such as OpenStreetMap [39] or country-specific, refined land cover data.

Data sets representing continuously varying properties across the landscape are projected, resampled and aggregated to match the gridded census buffer. These continuous data most often include elevation, slope, spatially explicit climate data, as well as mean annual NPP estimates. We also include the best available lights at night data, collected via satellite and mosaicked into a continuous dataset, subset to each country of interest [33]. Some studies have shown the effects of night light data may be exaggerated due to “light bloom” on neighboring pixels, effects from fires and gas production, and the difficulty of capturing dim but significant light associated with human settlement [40]. While these problems may be significant in a linear, parametric modeling context, the approach outlined here is more robust to these types of problems due to the flexible way in which model covariates can interact and influence one another. Furthermore, despite problems lights at night is a significant predictor in all models tested and so we include it for each country.

Another problem that arises is that many raster data sets are originally acquired at coarser spatial resolution than 100 m, as well as being clipped to overland areas (e.g. WorldClim, MODIS NPP). Once our raster covariates are resampled to match the rasterized census data and its buffer there may be areas with no data at the boundaries, especially on coastal boundaries. We use a simple nearest neighbor filling approach (the “nibble” tool in ArcGIS 10) to extend the edge of these data sets and fill any gaps prior to model estimation.

### Population Density Weighting Calculation

The data processing, model estimation and prediction for our algorithm is outlined in Fig. 1. For end-user population maps we use the finest level census data available during estimation and population redistribution, with counts adjusted using rural and urban growth rates to estimate population distribution for any particular year [25]. However, in these case studies we do not adjust census counts and instead add two steps to the process for comparison purposes (Fig. 1, orange areas). These steps involve aggregating census units to the next administration level up from the finest available. We use these aggregated counts during both estimation and prediction, which then allows us to compare sums from our pixel-level predictions with census counts from the original, fine-scale data. From this we estimate how accurate results may be across spatial scales. In this case study, we aggregated to administrative unit level four ("Division") for Kenya, and to unit level two ("Province") for Vietnam and Cambodia.

To attain covariate values for each aggregated administrative unit we calculated zonal means for each continuous dataset and counted class majorities for binary data sets. From this process we attained 38 covariates for VNM, 42 for KHM and 44 for KEN. These individual covariate values were merged with the census count for the aggregated census units, per respective country (Table 1).

Because our aim was to estimate population density for use as a dasymetric weighting layer we must estimate our Random Forest model with a population density “response” variable. Dasymetric redistribution in plain terms is a method to distribute a count representing a sum total (census unit population in this case) across an area (the administrative/census unit). Rather than distribute that count equally by area we can use one or more sets of data at a finer spatial scale than the administrative unit to unequally weight the redistribution. Ideally this weighting layer(s) better reflects underlying mechanisms contributing to the unequal distribution of the sum of interest.

We calculated the population density within each aggregated administrative unit, dividing the sum of census counts for the year of the census and dividing it by the area of the aggregated blocks. We then removed any census units with zero counts and log-transformed the density in order to create a more normal and even distribution of population density values with respect to our other covariates. Zero count census units are most often associated with protected areas or water bodies that are delineated in the original census data. In many census data sets these may represent a disproportionately large portion of the data and may bias estimation and prediction. In testing, eliminating zero count units and using a log transformed population density aids the Random Forest algorithm in finding good splits in the data as the relationship between distance-based covariates and population densities is in most cases more uniformly distributed.

### Weights Model Estimation by Random Forest

The population density and covariate aggregation values for each census unit were then used to create a Random Forest model [28] to predict log population density. Random Forest models are an ensemble, nonparametric modeling approach that grows a “forest” of individual classification or regression trees and improves upon bagging [41] by using the best of a random selection of predictors at each node in each tree [28, 29]. In many cases the predictive performance for Random Forests is on par with boosted regression trees [42] but have the advantage of having fewer tuning parameters. In our methodology this is especially important as the tuning can be automated as part of the fitting process. Furthermore, in the case where there are many correlated predictors or predictors with a large spectrum of informative value (e.g. some with a lot of information among many with very little) the Random Forest algorithm is attractive because the output from the forest growing algorithm can be used to estimate post-hoc variable importance measures.

Model estimation, fitting and prediction were all completed using the statistical environment R 2.15.3 [43] and the randomForest package 4.6–7 [29]. To streamline the prediction phase and reduce processing time we developed a multi-stage Random Forest estimation technique (see attached S2 File for further details). This technique first fit a series of models using the randomForest tuneRF function with all available covariates and the log population density of each census administrative unit as the response. The *tuneRF* function uses a step function to tune the Random Forest *mtry* parameter. This parameter determines the number of covariates to randomly select and choose from the best covariate for each node during the tree growing process. Prediction accuracies can be sensitive to the *mtry* parameter and *tuneRf* uses the minimization of out-of-bag-prediction error [29] as an objective function to select an appropriate value for *mtry*.

The next step in our algorithm is a very conservative covariate selection process for the resulting Random Forest. We perform this step in order to reduce the number of total covariates in the final Random Forest model which can significantly speed up per-pixel prediction. We use the resulting forest of trees grown for the tuned *mtry* value and extract covariate importance scores [29]. For any covariate that has a variable importance score of zero, which indicates that after random permutation of the covariate values no decline in mean squared error (MSE) of prediction is observed, we remove it from the list of potential covariates and re-run *tuneRF* with the reduced set of data. This is iterated until only positive importance scores remain for every covariate included in the modeling process. This usually converges in only one or two iterations and results in the minimum number of covariates that have even a small amount of predictive capacity, while eliminating covariates that are completely redundant or negatively impact prediction.

The only other tuning parameters required for Random Forest estimation are the number of trees to grow per forest and the number of observations to allow in terminal nodes. The latter controls the complexity of each individual tree in the forest by restricting the number of splits required to partition all of the observed response values with the randomly selected covariates. Trees are grown to a maximum size where terminal nodes contain at most the number of census units divided by 1000 (rounded to the nearest whole number) or a single observation when there are fewer than 1000. Predictions for the entire forest average the selected individual node by each tree in the forest. The sensitivity of prediction accuracy to these parameters is relatively low. Based on multiple experimental runs we observed that 500 trees were sufficient across all combinations and permutations of data to arrive at a stable, minimized out-of-bag (OOB) error of prediction [28]. Furthermore, we allowed the trees to grow maximally, which for typical dataset sizes in the regression context could introduce bias but with the number of trees chosen are mostly unbiased for final predicted log densities.

The resulting Random Forest is used to predict a country-wide, pixel-level map of log population densities (Fig. 1). The reduced set of covariate rasters, as arrived at through the variable importance selection algorithm, is stacked together into a single raster object. Then the Random Forest model trees and the covariate stack are distributed to one or more parallel processing environments and predictions are performed for every pixel within the country. Each tree in the forest of regression trees is used to predict a log population density for each pixel. From the collection of predictions for each pixel various summaries might be made. From experiments we showed that the best, most unbiased prediction was arrived at by taking the mean of all trees within the forest and back-transforming the log to arrive at an estimate of per-pixel population density. Medians and percentile ranges were also assessed as alternative approaches for prediction; however, the back-transformed mean consistently out-performed the alternative summary methods during validation. The resulting country-wise population density map was then used as a weighting layer for a standard dasymetric mapping approach as described for the AfriPop and AsiaPop data sets by Gaughan, et al. [19] and Linard, et al. [17]. The final national-level population data sets were then projected for 2010, 2015, and 2020 based on rural and urban growth rates estimated by the UN World Urbanization Prospects Database, 2012 version (UNPD) [4] using the following equation:

P_{2010}=P_{d}e^{rt}

where P_2010_ (P_2015_, P_2020_) is the required population for respective year, P_*d*_ is the population at the year of the input population data set, *t* is the number of years between the input data and the year being projected, and *r* is the urban or rural average growth rate. To assign the respective urban and rural growth rates, units were specified as “urban” if the population map pixel overlaps one classified as urban by the Schneider et al. [37, 38] product. If not, then these areas were assigned a “rural” class. Final population data sets include one version of data sets in which total population are adjusted to match UN national estimates and one set of data left unadjusted. In addition, data sets are created in which the individual cell values of the resulting population maps represent people per pixel and another with pixel values representing people per hectare.

### Accuracy Assessment and Comparison of Global Population Data sets

The final population maps produced from the one-level-up census aggregations were compared with those prepared using the GPW, GRUMP, and Afri/AsiaPop methodologies, as described by Gaughan, et al. [19]. Though comparable in many respects, insufficient information on input datasets and modeling methods are provided to enable replication of the LandScan methodology, so we exclude it from direct comparison. The individual cell values of the output population maps represent people per pixel. We summed the pixel values within each of the finer level census units for each country. These “predicted” sums were then compared with the observed census counts within each unit. Summary statistics were calculated for each methodology, including root mean square error (RMSE), the RMSE divided by the mean census unit count (%RMSE) and the mean absolute error (MAE). Together these statistics are used to compare the relative strength of predictive value that each methodology exhibits, when scaling down predictions.

Though many prediction studies use an approach that creates a “hold-out,” random sample of the available data to use only in validation, the seminal literature on the Random Forest algorithm indicates both empirically and theoretically that it is unnecessary if the goal is to estimate general prediction error (Section 3.1 of Breiman, 2001) [28]. The reason is that the Random Forest algorithm employs “bagging” [41] for each tree in the forest. This involves selecting a random sample of the training data, the size of the original data, by sampling with replacement. This random sample is then used to estimate the individual tree splits after which the “out-of-bag” (OOB) error is calculated (the mean squared error) for those observations in the original training data which were not part of the random sample. Since bagging occurs for every tree in the forest the mean OOB error is a robust measurement of error since errors are estimated for samples that were not part of the estimation process in individual trees. But bagging does not reduce the predictive capacity of the model since un-sampled observations for any one tree will likely be sampled in other trees during the creation of the Random Forest. It should be noted that since we are predicting to the pixel level the OOB error estimates do not necessarily represent expected errors of prediction at the smaller spatial scale, nor even errors in estimates aggregated to the census unit. However, by considering the estimated OOB error rates at the census unit level, which range from 83% variance explained for Kenya, and 93% for both Cambodia and Vietnam (see metadata reports attached as S1 File), as well as the one-level-up predictions versus finer level observations, we conclude that the accuracies in Random Forest predicted weighting layers result in improved accuracies of the final, redistributed population maps.

## Results

### Population data sets

**Fig 2:**
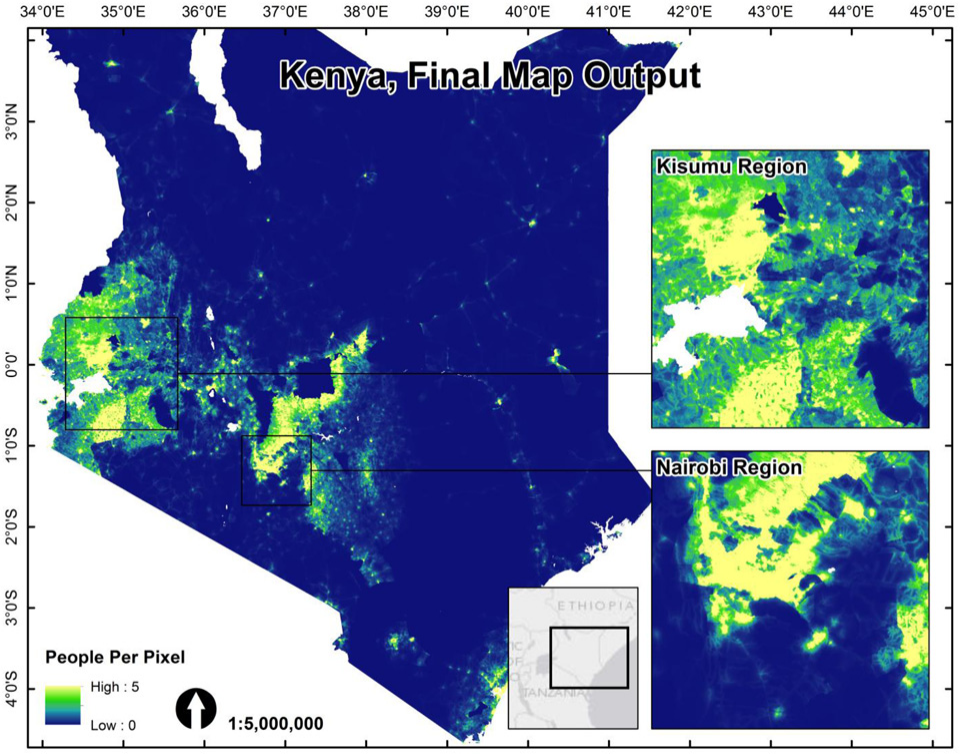
The final redistributed population map for the Kenyan 1999 census data. Both census counts and the Random Forest predicted weighting layer for dasymetric redistribution were based on the finest level administrative units (Level 4) where a complete, country-wide coverage was available.

Final end-user 2010, 2015 and 2020 population data sets were generated for each country with an example of the final 2010 output for Kenya shown in Fig. 2 (please find Cambodia, Vietnam, and Kenya metadata reports and population maps attached as S1 File). We also produced adjusted maps that match UN national total population estimates for each year according to the methodology described by Linard, et al. [17]. All maps have a spatial resolution set at 8.33 x 10^–4^ degrees latitude/longitude and represent the number of people per grid cell.

### Random Forest Output

**Fig 3:**
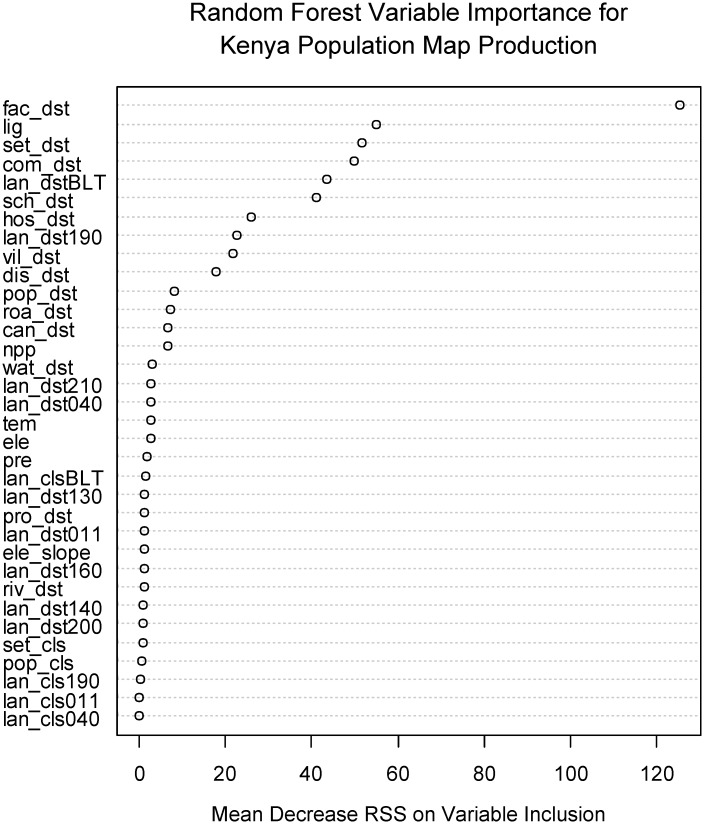
Variable importance for Random Forest regression, presented as the mean decrease in residual sum of squares when the variable is included in a tree split. The model including variables here was used to produce the density weighting layer for the dasymetrically distributed population map in Fig. 2. Variable names are defined and described in Table 1.

The Random Forest model performs substantially better than several other commonly used, freely available approaches for dasymetric mapping at country-level scales. An assessment of which of the ancillary data covariates are important for accurately estimating population density at the census unit level is produced by the Random Forest algorithm (see the third figure in each metadata report attached as S1 File). During the variable selection phase of the algorithm, the values of variable importance may fluctuate as the number of covariates is reduced. However, the relative ranking is quite stable among the top covariates. For Kenya, distance to health facility is by far the most important predictor (Fig. 3) for reducing the amount of variability left in the log population densities of the training census data. This indicates that this ancillary dataset is extremely valuable, even more than the distance-to-built land cover which is typically extremely important (see metadata reports in S1 File). This is likely due to the comprehensive and detailed nature of the health facility dataset [44].

### Accuracy Assessments

**Fig 4:**
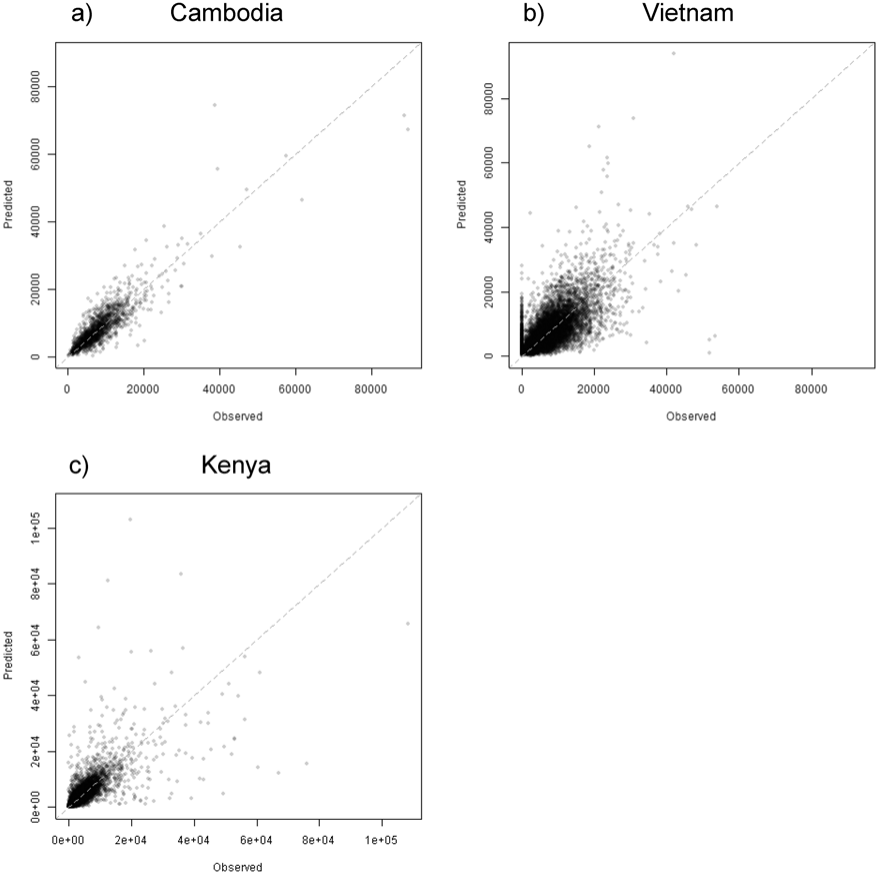
Observed census counts plotted from the finer census administrative units versus the summed grid cell values from the population map estimated using coarser administrative units for a) Cambodia, b) Vietnam, and c) Kenya. Dotted lines in each figure represent the 1:1 line and prediction error is stated for each country by the RMSE, %RMSE and MAE values.

The accuracies of the population maps which were constructed using coarser scale administrative units are presented in Fig. 4 and in Table 2. For Cambodia (Fig. 4a) there appears to be very little bias in prediction error across the range of observed census values. The calculated RMSE, % RMSE and MAE indicate that the performance of the new method is better than any of the other tested approaches (Table 2). Though not as tight as the relationship for Cambodia (Fig. 4a), the observed vs. predicted plot for Vietnam (Fig. 4b) shows that the RF methodology is not particularly biased for observed values larger than zero. Where observed census counts are zero, however, the methodology will predict a range of values, which indicates there may still be some refinement possible to better predict when census units include low or zero counts.

**Table 2:** Accuracy assessment results for the RF, Afri/AsiaPop, GRUMP and GPW modeling methods for Cambodia, Vietnam and Kenya.

Country	Method	RMSE	% RMSE	MAE
Cambodia	RF	3209.40	38.84	2026.73
	AsiaPop	3834.51	46.40	2494.32
	GRUMP	6767.39	81.89	3889.39
	GPW	6794.88	82.22	4021.41
Vietnam	RF	4367.00	61.96	2778.80
	AsiaPop	4943.31	70.13	3007.04
	GRUMP	6523.77	92.56	3771.64
	GPW	7081.76	100.47	3844.47
Kenya	RF	3956.74	91.36	1685.82
	AsiaPop	5208.79	120.28	2184.64
	GRUMP	6294.61	145.35	2383.64
	GPW	6327.80	146.11	2304.70

Two different error assessment methods are presented: root mean square error (RMSE), also expressed as a percentage of the mean population size of the administrative level (% RMSE); and the mean absolute error (MAE).

**Fig 5:**
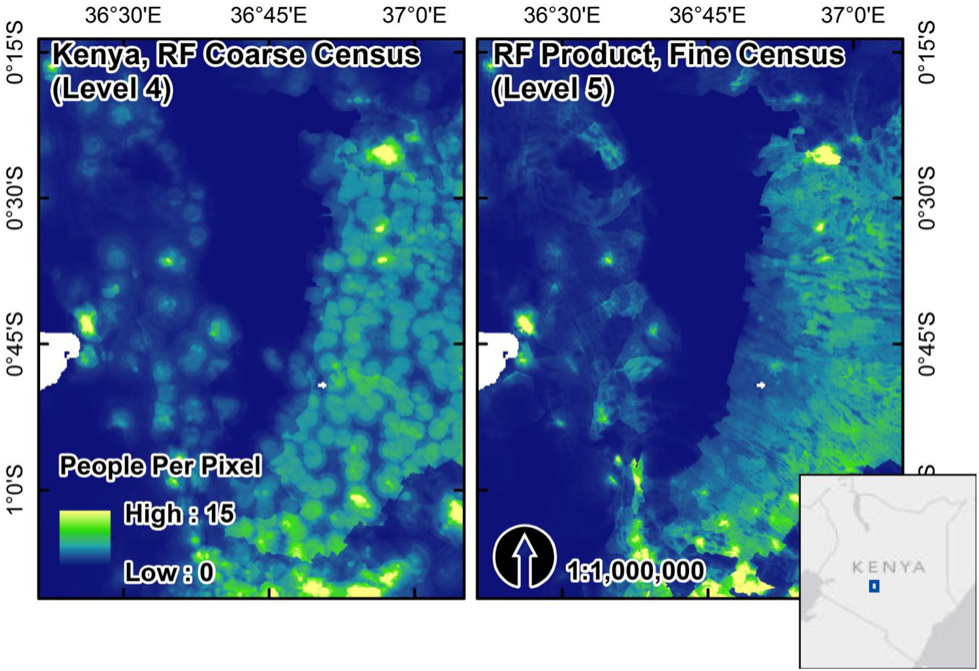
A visual comparison of Kenyan population maps for census data in 1999 produced at coarser administrative unit (Level 4) and finer-scale administrative unit (Level 5). The difference illustrates the finer gradations of RF model predictions for the density weighting layer when there are larger ranges of observed population densities present in training data (N = 505 for Level 4, N = 6622 for Level 5).

The use of finer-level administrative units in the algorithm increases the ability of the RF model to predict population density at smaller scales, which takes better advantage of useful ancillary datasets due to the increased variability explained in observed population counts. We show this visually by estimating the RF model and generating population maps for Kenya using finer level census units (Administrative Level 5, or ‘sublocation’). These finer scale results show a smoother, more continuous redistribution of population versus the model estimated with coarser level administrative units (Administrative Level 4) (Fig. 5).

**Fig 6:**
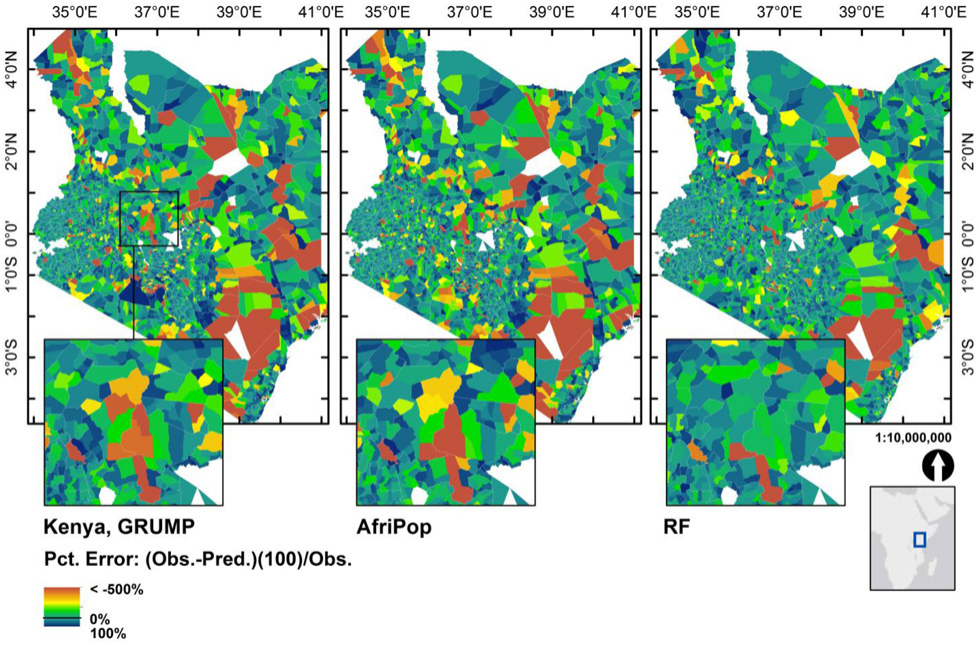
For Kenya we compared the summed predictions of population maps estimated using coarse census data (Level 4, “Division” level) to population counts at Level 5 (finer scale) units. We present prediction errors (observed minus predicted) as percentage values of the observed Level 5 census counts. The RF approach results in far fewer census units with extreme over predictions (negative percent residuals in yellows and oranges) and under predictions (positive percent residuals in dark blues).

For validation purposes, we used the predicted population maps from Kenya estimated with the Level 4 administrative unit census data, and compared the percent residual, (observed—predicted) / observed) x 100) for each census unit summed within the admin Level 5 (finer scale) units (Fig. 6). A negative percent residual represents an over prediction, while the scale is bounded by 100% for the case where you predict zero but there were actually people counted within that census unit. The distribution of percent residuals shows that the RF approach results in fewer extreme prediction errors across the entire country. Furthermore, there are fewer extremes in both large and small census units, indicating that the percent error is relatively consistent for both large and small population counts.

## Discussion

### Main Summary

**Fig 7:**
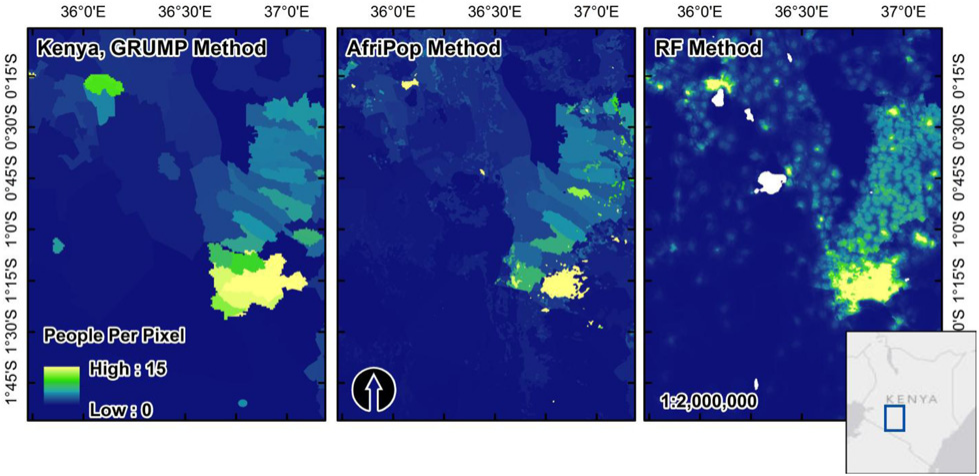
Visual comparisons of GRUMP, AfriPop and the RF-based population map from this study. Though this region northwest of Nairobi, Kenya is not a highly populated region this figure shows the results of the more detailed RF weighting layer versus the use of just urban areas (GRUMP) and land cover plus urban areas (AfriPop). The distinct edges in estimated people per pixel between census units are almost eliminated by the RF approach and it achieves greater consistency in predicted population density after census count redistribution.

The population mapping method presented here creates a dasymetric weighting scheme using an iterative Random Forest (RF) model with multiple ancillary data sources. By using this flexible weighting algorithm we improve upon existing, freely available population mapping approaches and increase our ability to provide updated population maps for countries with census and ancillary data at spatial scales relevant to country-level population density maps. We compared this approach to other population map production algorithms. The AfriPop and AsiaPop approaches use a weighting scheme calculated from class-based combinations of land cover, urban/rural built area delineation and climate zone information [17, 19]. By incorporating such information into the AfriPop/AsiaPop weighting methodology, census counts are distributed more densely in human-modified areas than GRUMP and GPW, thereby increasing the accuracy of final the products (Fig. 7). The RF methodology we outline here improves upon this weighting algorithm by incorporating data sets other than just land cover and climate zone, including distance- to health facilities, schools, roads and many other features that may be good predictors or proxies for human population presence (Table 1). By including these features in the updated RF weighting algorithms we create population maps that are not only more accurate than GPW, GRUMP and AfriPop/AsiaPop (Table 2), but also represent variability in population density as it relates to multiple biophysical and social features across the landscape.

### Methodology Considerations

One of the strengths of the Random Forest algorithm is the ability to incorporate many covariates with a minimum of tuning and supervision. These covariate data sets include night-time lights (e.g. Suomi NPP Visible Infrared Imaging Radiometer Suite (VIIRS)-derived data), topography (e.g. USGS SRTM-derived HydroSheds, ASTER DEM products), land cover (e.g. Landsat-derived classified data sets) and climatic information (e.g. WorldClim). A broad range of country-specific data may also be incorporated to further refine any modeling effort. These data may include road and river networks, population center and building locations, protected area boundaries, and demography, among others. The primary challenge presented by combining these disparate data in any modeling effort lies in the multiple scales of measurement, often high correlation among data sets, the presence of highly nonlinear interactions and a mix of continuous and discrete distributional characteristics.

The Random Forest model estimated for Kenya (Fig. 4c) exhibits a slight tendency to over-predict at low population values and exhibits a wider spread around the 1:1 line. However, because Kenya has a much larger range of census values with many smaller administrative units it exhibits the lowest MAE among the case study countries but the highest % RMSE. It remains a difficult country for which to predict population distributions at a very fine scale. But because the available census data are so fine, the dasymetric mapping approach for the final end-user product will still have high accuracy as the census counts “anchor” the end-user product predictions to observed values at a smaller spatial scale than for many other countries. This “anchor” effect minimizes the bias introduced by the “Ecological Fallacy” of using a model estimated with aggregated data, in this case the census unit, to predict at a much finer spatial scale. By redistributing population data within each census unit to predicted population density, estimates are guaranteed to at least be accurate when aggregated back to the census unit level.

### Context

When developing models that incorporate multiple data sources there are two primary approaches. The first relies on statistical models for predicting population density directly from multiple covariates [26]. These are often used at small geographic scales (sub-regional, metropolitan or county-level) and rely on highly detailed population count or density information for model estimation and often place restrictive demands on distributional requirements and assumptions. The second relies on creating a population redistribution weighting scheme that is combined with areal census counts, and with a dasymetric approach, spreads those counts across the area they represent as a function of the weights within each census area. This approach can be used at large scales (i.e. country and continent scale) and can vary widely in the sophistication of the weighting scheme used. The most well-known large-scale projects using ancillary data sources are LandScan [13], AfriPop [5, 17] and AsiaPop [19].

We are challenged to incorporate the vast and increasing amounts of spatial data being regularly produced on features that influence population distributions (e.g. more detailed and accurate road locations, settlement centroids, building delineations). To take advantage of these data a flexible modeling approach capable of dealing with collinearity and nonlinearity in variable associations is required. The outlined RF methodology is highly resistant to over-fitting training data, exhibits a good balance of bias-variance optimization and generates estimates of both variable importance and model uncertainty as a byproduct of the cross-validation inherent to the fitting process [28, 29]. Though in other applications it performs slightly worse than recent gradient boosting algorithms [45] the RF algorithm has fewer tuning parameters which makes it preferable in the semi-automated map production process we have developed here. Enabling a more semi-automated mapping approach is preferred here for generating cross-national population surfaces with minimal country-specific tuning, thus providing updated population data sets in a more timely and effective manner.

Several features of the RF algorithm and their implications for the population distribution weighting layer warrant additional discussion. We use the natural log of the observed, census unit population densities as the response variable for RF model estimation. We found that by transforming the response variable prior to RF model fitting we consistently achieved higher prediction accuracies in the validation process we describe (Fig. 1, Table 2). Transformation of the covariates will not affect the explanatory power or fitting process of the RF algorithm [28] because splitting criteria at each node are evaluated along the entire range of bagged covariates. Therefore, any transformation of a covariate that does not affect the covariate’s rank order will not have an overall effect on model fitting or predictions. However, transformations of the response variable can have a significant impact on model fitting and performance because the range and features of the response distribution affect the values calculated for the splitting criteria objective function (sum of squared deviations for predictions in the RF regression case).

We evaluated several different transformations of the observed census-unit population density including square root, log_10_, and others. The natural log transformation resulted in the largest increases in prediction accuracy but have the consequence of requiring the removal of census-units with zero population densities from the training data prior to fitting. The gains are likely seen from the decreased impacts of prediction errors for large observed population densities, thus prioritizing prediction accuracy for lower- and mid-range population density areas. But predicting log-population density and back-transforming results in a dasymetric density weighting layer that has no cells with zeroes. This means that at least a small fraction of census population numbers will be spread to every cell of the resulting population map, except for those cells classified as water or missing in the land cover layer for which we define population density to be zero, or for areas that are outside the census unit boundaries. This may have implications for certain applications of the population maps where whole counts are preferred, however, statistical or spatial reassignment on a cell-by-cell basis could be performed by the end-user to alleviate this issue. Alternatives to the natural log transformation will be explored in future algorithm development to allow for zero population predictions and further refinement of the prediction accuracy. These could include a two-stage modeling approach, predicting zero or non-zero first and following up with the algorithm as we describe here for areas with predicted non-zero population density.

Another feature of the RF algorithm for regression that has implications for population maps and dasymetric weighting is that RF regression predictions are limited to the range of values present in the training observations. Therefore, no extrapolation beyond the bounds of the population densities observed in the original training data is possible. Within the census-unit level we know that population density may be spatially heterogeneous, especially as census-unit sizes increase, but even within small census unit areas. Our primary assumption is that RF predictions at the pixel level, even limited to the range of observed population densities at the census-unit level, will accurately represent the spatial heterogeneity within the census-unit for more accurate dasymetric redistribution of population counts. This assumption is more likely to be true as you decrease the size of the census units with which the RF model is estimated, resulting in finer gradations of model predictions across the ranges of covariates used (Fig. 5). Fortunately, for countries with coarse census data (large census units relative to the country size and population density within them) the RF model will degrade gracefully to predictions of mean population density and bias in predictions will tend to be minimized in favor of less specificity. In other words the RF algorithm will do no worse than model approaches with less ancillary data.

However, the RF approach we outline here also offers a potential solution to the coarse census data problem. The demonstrated strength of the RF algorithm for countries with fine-scale census data, like those presented here, can be leveraged for those countries with coarser data. By combining the forests produced from one or more countries with similar constraints on population distributions and available ancillary data, we can use the combined model to create a predicted density weighting layer for countries that have coarse census data. Furthermore, as more census data become available for single or multiple countries within a region, we can further refine RF predictions by sampling from and combining trees and forests which may yield better prediction accuracies than using a single RF model parameterized using coarse-scale census data.

Despite these caveats and considerations, the application of the Random Forest algorithm allows for increased detail in the final population redistribution, minimizing artifacts in the final population dataset. Data sets are more accurate than other large-scale regional and global scale population data sets (Table 2) and produce smoother, more continuous and ‘realistic’ looking surfaces as a product of the increased amount of ancillary data used in the model process [26]. Variability at finer scales is an important consideration that the RF methodology incorporates by using a significant number of ancillary datasets that inform the final population redistribution process for each country. We show that these ancillary datasets, collected regionally and per-country may be incorporated into our flexible modeling framework more easily than with other approaches and allow us to quickly produce finer-scale, mapped population distribution products than previously available.

## Conclusions

We present a semi-automated, dasymetric model for mapping human population distribution that provides a timely resource used for measuring impacts of population growth. Increased accuracy of projected changes in population patterns is important for policy and planning initiatives at regional to global scales and results from this study provide a methodology for generating highly accurate national-scale distributions of population data. By using a Random Forest algorithm to determine population density weightings, the model algorithm is more flexible in handling multiple covariates of both a discrete and continuous nature. The general methodology increases the sophistication of the weighting scheme first introduced in the AfriPop and AsiaPop dasymetric mapping projects [5, 17–19] by introducing a suite of ancillary datasets that enable finer-scale variability in population redistribution. Population datasets for 2010, 2015, and2020are freely available for download from the WorldPop Project (http://www.worldpop.org.uk/).

## Supporting Information

S1 File Population Maps, Metadata Reports and KML Files. For each country we produce population maps for the year the census data were collected (e.g. Fig. 5). In addition, population maps are adjusted to match UN estimates of growth for rural and urban areas to produce estimated population maps for 2010 and 2015. These estimates are also adjusted to match UN total population estimates for each country. The methodology for making these adjustments is detailed by Linard et al. [17]. In addition to the population maps themselves, the algorithm self-documents by creating a metadata report. This report includes not only information on the covariates included in each country’s model but also the Random Forest fitting information. Last, the population map for the census data year is tiled and saved into a Google Earth KML/KMZ file for easy overlay on top of high-resolution imagery. Examples of the metadata reports and KMZ files are attached with this manuscript as for Cambodia (KHM), Vietnam (VNM) and Kenya (KEN).
(ZIP)

S2 File Technical Fitting Details of the Random Forest Algorithm and Source Code. Though the randomForest package [29] provides the functionality to fit a model with an arbitrarily large number of covariates and observations (limited only by memory and disk space) a limiting feature of our approach is the time spent during the prediction phase. During testing with covariates from Kenya we found that decreasing the number of predictors from 44 to 16 during the final forest growing stage and using the reduced forest for prediction over millions of pixels can result in time reduction of 1–2% per predictor decreased. For a prediction running in parallel, with a country the size of Kenya, on a standard dual core laptop or desktop processor running at 2.5 GHz this can reduce prediction times by as much as five hours. This increase in efficiency comes with little to no trade-off in out-of-bag prediction accuracy. In practice model estimates are performed on a multi-core machine and run in parallel fashion over more than two cores, with the entire process running from data pre-processing to completion on the order of hours to as much as a day for very large countries. The data reduction method is attached, packaged as an R code snippet with included data and covariates shapefile to reproduce the method described. In the attached source code please assume that *y_data* is a vector containing log transformed population densities for each census unit in the data set. Also assume that *x_data* is a data.frame containing a row for each census unit and columns for each aggregated covariate (continuous measurements like distances or proportions are averaged, while categorical covariates are mode aggregated). The sample shapefile provided includes covariate data aggregated for Cambodia and the compressed R data frame files (for *x_data* and *y_data*) used with the included code snippet will recreate the Random Forest model estimation used for the final Cambodia maps presented in the paper are attached. All of the initial tuning parameters are estimated as a simple function of number of observed census units, while the *tuneRF()*function is used to find the optimal number of covariates to randomly select on at each branch point. These Random Forest objects are then used in a parallel fashion to generate per-pixel predictions of population density. These per-pixel predictions make up the layer used to dasymetrically distribute census-level population counts.
(ZIP)

S3 File Additional Accuracy Test Using Total Country-wide Population Redistribution. We illustrate the utility of the Random Forest methodology and the prediction density layer it produces by summing all census units into a single country-wide population count. We then distribute the total population number according to the relative weight for each pixel in our prediction layer, just as we would for individual census units. Doing this for Cambodia (S3 File) and comparing the distribution to results where individual census unit counts were used as the units for dasymetric redistribution (Fig. 4a) illustrates the utility of the prediction layer as a whole. The results show that though we lose some predictive power (illustrated by the greater spread around the 1:1 line) we are not increasing bias in our estimates (illustrated by the linear trend of the observed vs. predicted values along the 1:1 line). The resulting RMSE of 4672 indicates that even when eliminating the “anchor” effect of distributing census unit counts, the Random Forest methodology still outperformed the approaches used by GRUMP and GPW methods, even though they are both using finer-level census data to anchor their predictions.
(TIF)
