# Supplementary material for: Disaggregating Census Data for Population Mapping Using Random Forests with Remotely-Sensed and Ancillary Data
Source: PLoS One. 2015 Feb 17;10(2):e0107042. doi: 10.1371/journal.pone.0107042 (PMC4331277; doi:10.1371/journal.pone.0107042)
Supplement: S1 File — For each country we produce population maps for the year the census data were collected (e.g. Fig. 5). In addition, population maps are adjusted to match UN estimates of growth for rural and urban areas to produce estimated population maps for 2010 and 2015. These estimates are also adjusted to match UN total population estimates for each country. The methodology for making these adjustments is detailed by Linard et al. [17]. In addition to the population maps themselves, the algorithm self-documents by creating a metadata report. This report includes not only information on the covariates included in each country’s model but also the Random Forest fitting information. Last, the population map for the census data year is tiled and saved into a Google Earth KML/KMZ file for easy overlay on top of high-resolution imagery. Examples of the metadata reports and KMZ files are attached with this manuscript as for Cambodia (KHM), Vietnam (VNM) and Kenya (KEN). (ZIP) [file pone.0107042.s001.zip › KEN_metadata.html]

Kenya Population Map Metadata Report


# Kenya Population Map Metadata Report

## Prediction Weighting Layer Used in Population Redistribution

The data presented below represent the predicted number of people per ~100 m pixel as estimated using the random forest (RF) model as described in Stevens, et al. (In Press). The following pages contain a description of the RF model and its covariates, their sources and any metadata collected for each covariate. The prediction weighting layer is used to dasymetrically redistribute the census counts and project counts to match estimated populations based on UN estimates for the final population maps provided by AfriPop, AsiaPop and AmeriPop.

## Kenya Census Data and Observed Population Density

These data are the population density values used to estimate the RF model used to create the prediction weighting layer you see above. Values represent population density as measured by people per hectare and calculated from population counts within each census unit. These values are used as the dependent variable during model estimation.

### Kenya Census Data, 1999, Admin-level 5

**Folder:** Census  
**File Name:** KEN\_census\_1999\_sublocations\_topo.shp  
**Source:** Kenya National Bureau of Statistics, acquired by Tatem, et al. for use in AfriPop data products.  
**Description:** These census data were acquired for use as a disaggregation layer for more-recent census data for AfriPop. It is used here on its own to produce a disaggregated population map for 1999 because it is the finest level census data available. Required fields for map production are ADMINID and ADMINPOP.  
**Class:** polygon  
**Derived Covariates:**  
area, buff, zones,

```
class       : SpatialPolygonsDataFrame 
nfeatures   : 6624 
extent      : -66764, 823501, -517009, 605783  (xmin, xmax, ymin, ymax)
coord. ref. : NA 
nvariables  : 50
```

---

## Random Forest Model and Diagnostics

These output and figures outline the estimated RF model that is used to predict the population density weighting layer. The model is fitted to the population density values for the preceding census data using covariates aggregatedfrom the ancillary data sources summarized following the model diagnostics.

```
Call:
 randomForest(x = x_data, y = y_data, ntree = popfit$ntree, mtry = popfit$mtry,      nodesize = length(y_data)/1000) 
               Type of random forest: regression
                     Number of trees: 500
No. of variables tried at each split: 8

          Mean of squared residuals: 0.67
                    % Var explained: 83
```

## Covariate Metadata

### Kenya Classified Land Cover

**Folder:** Landcover  
**File Name:** KEN\_gc\_reclass\_0.0008333\_rurb\_8bit.img  
**Source:** GlobCover, 300m  
**Description:** Landcover from the GlobCover product, reclassified to match AfriPop coding and eventually broken down into binary classifications by aggregated land cover type (see Linard, et al., 2010 and Gaughan, et al. 2013 for category information).  
**Class:** raster  
**Derived Covariates:**  
prp011, cls011, dst011, prp040, cls040, dst040, prp130, cls130, dst130, prp140, cls140, dst140, prp150, cls150, dst150, prp160, cls160, dst160, prp190, cls190, dst190, prp200, cls200, dst200, prp210, cls210, dst210, prp230, cls230, dst230, prp240, cls240, dst240, prp250, cls250, dst250, prpBLT, clsBLT, dstBLT,

```
class       : RasterLayer 
dimensions  : 11263, 8911, 100364593  (nrow, ncol, ncell)
resolution  : 100, 100  (x, y)
extent      : -66765, 824335, -519218, 607082  (xmin, xmax, ymin, ymax)
coord. ref. : +proj=utm +zone=37 +datum=WGS84 +units=m +no_defs +ellps=WGS84 +towgs84=0,0,0 
data source : D:\Documents\Graduate School\Research\Population\Data\RF\data\KEN\Landcover\Derived\landcover.tif 
names       : landcover 
values      : 0, 240  (min, max)
attributes  :
       ID OID Value    Count
 from:  0   0    11 19254933
 to  :  9   9   240     8562
```

---

### MODIS 17A3 2010 Estimated Net Primary Productivity, 1km

**Folder:** NPP  
**File Name:** KEN\_gc\_reclass\_0.0008333\_rurb\_8bit.img  
**Source:** United States Geological Survey (USGS)  
**Description:** MODIS 17A3 version-55 derived estimates of net primary productivity for the year 2010, estimated for 1km pixel sizes and subset and resampled to match the available land cover and final population map output requirements.  
**Class:** raster  
**Derived Covariates:**  
,

```
class       : RasterLayer 
dimensions  : 11263, 8911, 100364593  (nrow, ncol, ncell)
resolution  : 100, 100  (x, y)
extent      : -66765, 824335, -519218, 607082  (xmin, xmax, ymin, ymax)
coord. ref. : +proj=utm +zone=37 +datum=WGS84 +units=m +no_defs +ellps=WGS84 +towgs84=0,0,0 
data source : D:\Documents\Graduate School\Research\Population\Data\RF\data\KEN\NPP\Derived\npp.tif 
names       : npp 
values      : 0, 22341  (min, max)
attributes  :
          ID Rowid    COUNT
 from:     0     0 11089043
 to  : 22341 19433       90
```

---

### Suomi NPP VIIRS-Derived 2012 Lights at Night, 15 arc-second

**Folder:** Lights  
**File Name:** DEFAULT: VIIRS 2012  
**Source:** http://ngdc.noaa.gov/eog/viirs/download\_viirs\_ntl.html  
**Description:** These 'Lights at Night' data were derived from imagery collected by the Suomi National Polar-orbiting Partnership (NPP) Visible Infrared Imaging Radiometer Suite (VIIRS) sensor. Data were collected in 2012 on moonless nights and though background noise associated with fires, gas-flares, volcanoes or aurora have not been removed it represents the best-available data for night-time light production.  
**Class:** raster  
**Derived Covariates:**  
,

```
class       : RasterLayer 
dimensions  : 11264, 8912, 100384768  (nrow, ncol, ncell)
resolution  : 100, 100  (x, y)
extent      : -66865, 824335, -519218, 607182  (xmin, xmax, ymin, ymax)
coord. ref. : +proj=utm +zone=37 +datum=WGS84 +units=m +no_defs +ellps=WGS84 +towgs84=0,0,0 
data source : D:\Documents\Graduate School\Research\Population\Data\RF\data\KEN\Lights\Derived\lights.tif 
names       : lights 
values      : -0.36, 182  (min, max)
```

---

### WorldClim/BioClim Mean Annual Temperature 1950-2000, 30 arc-second

**Folder:** Temp  
**File Name:** DEFAULT: BIO1  
**Source:** http://www.worldclim.org/current  
**Description:** WorldClim/BioClim 1950-2000 mean annual precipitation (BIO12) and mean annual temperature (BIO1) estimates (Hijmans et al., 2005) were downloaded, mosaicked and subset to match the extent of our land cover data for the mapping of this region.  
**Class:** raster  
**Derived Covariates:**  
,

```
class       : RasterLayer 
dimensions  : 11264, 8912, 100384768  (nrow, ncol, ncell)
resolution  : 100, 100  (x, y)
extent      : -66865, 824335, -519218, 607182  (xmin, xmax, ymin, ymax)
coord. ref. : +proj=utm +zone=37 +datum=WGS84 +units=m +no_defs +ellps=WGS84 +towgs84=0,0,0 
data source : D:\Documents\Graduate School\Research\Population\Data\RF\data\KEN\Temp\Derived\temp.tif 
names       : temp 
values      : -50, 296  (min, max)
attributes  :
        ID OID Value Count
 from:   0   0  -290     1
 to  : 609 609   320    31
```

---

### WorldClim/BioClim Mean Annual Precipitation 1950-2000, 30 arc-second

**Folder:** Precip  
**File Name:** DEFAULT: BIO12  
**Source:** http://www.worldclim.org/current  
**Description:** WorldClim/BioClim 1950-2000 mean annual precipitation (BIO12) and mean annual temperature (BIO1) estimates (Hijmans et al., 2005) were downloaded, mosaicked and subset to match the extent of our land cover data for the mapping of this region.  
**Class:** raster  
**Derived Covariates:**  
,

```
class       : RasterLayer 
dimensions  : 11264, 8912, 100384768  (nrow, ncol, ncell)
resolution  : 100, 100  (x, y)
extent      : -66865, 824335, -519218, 607182  (xmin, xmax, ymin, ymax)
coord. ref. : +proj=utm +zone=37 +datum=WGS84 +units=m +no_defs +ellps=WGS84 +towgs84=0,0,0 
data source : D:\Documents\Graduate School\Research\Population\Data\RF\data\KEN\Precip\Derived\precip.tif 
names       : precip 
values      : 172, 2624  (min, max)
attributes  :
         ID  OID Value   Count
 from:    0    0     0 1157797
 to  : 9586 9586 11401       1
```

---

### Road Network

**Folder:** Roads  
**File Name:**  
**Source:** Kenya Bureau of Statistics, acquired by Andrew J. Tatem  
**Description:** This is a detailed road layer available country-wide and provided by the Kenya Beureau of Statistics to project members.  
**Class:** linear  
**Derived Covariates:**  
dst,

```
class       : SpatialLinesDataFrame 
nfeatures   : 148897 
extent      : -62788, 818799, -515486, 585666  (xmin, xmax, ymin, ymax)
coord. ref. : NA 
nvariables  : 51
```

---

### River Network

**Folder:** Rivers  
**File Name:** DEFAULT: hydro/watrcrsl  
**Source:** National Geospatial-Intelligence Agency (NGA), http://geoengine.nga.mil/geospatial/SW\_TOOLS/NIMAMUSE/webinter/rast\_roam.html  
**Description:** The VMAP0 data area downloaded as separate files, grouped roughly by continent, and merged into individual shapefiles for subsetting and further processing for population mapping efforts. These data were obtained directly from the original VMAP0 data sources provided by the NGA and pre-processed using Military Analyst in ArcGIS 10.0.  
**Class:** linear  
**Derived Covariates:**  
dst,

```
class       : SpatialLinesDataFrame 
nfeatures   : 3008 
extent      : -68015, 828354, -519028, 610624  (xmin, xmax, ymin, ymax)
coord. ref. : NA 
nvariables  : 8
```

---

### Populated Places, Satellite-Derived

**Folder:** Populated  
**File Name:**  
**Source:** Tatem, A. J., Noor, A. M., & Hay, S. I. (2004). Defining approaches to settlement mapping for public health management in Kenya using medium spatial resolution satellite imagery. Remote Sensing of Environment, 93(1-2), 42�52. doi:10.1016/j.rse.2004.06.014  
**Description:** These data were created as described in the Tatem, et al. paper (2004) and derived from Landsat Thematic Mapper ™ and Japanese Earth Resources Satellite-1 (JERS-1) synthetic aperture radar (SAR) imagery at a 40 m nominal resolution.  
**Class:** polygon  
**Derived Covariates:**  
merged, cls, dst, prp,

```
class       : SpatialPolygonsDataFrame 
nfeatures   : 1660 
extent      : -47506, 818239, -515779, 466985  (xmin, xmax, ymin, ymax)
coord. ref. : NA 
nvariables  : 1
```

---

### Inland Waterbodies

**Folder:** Waterbodies  
**File Name:** DEFAULT: hydro/watrcrsl  
**Source:** National Geospatial-Intelligence Agency (NGA), http://geoengine.nga.mil/geospatial/SW\_TOOLS/NIMAMUSE/webinter/rast\_roam.html  
**Description:** The VMAP0 data area downloaded as separate files, grouped roughly by continent, and merged into individual shapefiles for subsetting and further processing for population mapping efforts. These data were obtained directly from the original VMAP0 data sources provided by the NGA and pre-processed using Military Analyst in ArcGIS 10.0.  
**Class:** polygon  
**Derived Covariates:**  
cls, dst, prp,

```
class       : SpatialPolygonsDataFrame 
nfeatures   : 275 
extent      : -76649, 784380, -505933, 554558  (xmin, xmax, ymin, ymax)
coord. ref. : NA 
nvariables  : 8
```

---

### Protected Areas

**Folder:** Protected  
**File Name:** DEFAULT: WDPAfgdb\_Sept2012.gdb  
**Source:** World Database on Protected Areas, Downloaded September, 2012, UNEP, http://www.wdpa.org, http://protectedplanet.net  
**Description:** These data are compiled by UNEP and distributed via the Protected Planet website. All protected areas were downloaded regardless of International Union for Conservation of Nature (IUCN) or any other designation, so they include sanctuaries, national parks, game reserves, World Heritage Sites, etc.  
**Class:** polygon  
**Derived Covariates:**  
cls, dst, prp,

```
class       : SpatialPolygonsDataFrame 
nfeatures   : 213 
extent      : -60520, 794358, -524625, 615783  (xmin, xmax, ymin, ymax)
coord. ref. : NA 
nvariables  : 26
```

---

### Elevation and Derived Slope, 3 second

**Folder:** Elevation  
**File Name:** DEFAULT: Void-Filled DEM.gdb  
**Source:** HydroSHEDS Void-Filled DEM (Lehnert, et al., 2006), http://hydrosheds.cr.usgs.gov/dataavail.php  
**Description:** The HydroSHEDS data are the result of an effort to provide a globally consistent dataset consisting of NASA's Shuttle Radar Topography Mission (SRTM) data and have been processed, void-filled and corrected for use at large scales.  
**Class:** raster  
**Derived Covariates:**  
, slope,

```
class       : RasterLayer 
dimensions  : 11264, 8912, 100384768  (nrow, ncol, ncell)
resolution  : 100, 100  (x, y)
extent      : -66865, 824335, -519218, 607182  (xmin, xmax, ymin, ymax)
coord. ref. : +proj=utm +zone=37 +datum=WGS84 +units=m +no_defs +ellps=WGS84 +towgs84=0,0,0 
data source : D:\Documents\Graduate School\Research\Population\Data\RF\data\KEN\Elevation\Derived\elevation.tif 
names       : elevation 
values      : -18, 5875  (min, max)
```

---

### Built Areas, Satellite-Derived, 100 m

**Folder:** Built  
**File Name:** ksm-imagine3\_100m.img  
**Source:** Tatem, A. J., Noor, A. M., & Hay, S. I. (2004). Defining approaches to settlement mapping for public health management in Kenya using medium spatial resolution satellite imagery. Remote Sensing of Environment, 93(1-2), 42�52. doi:10.1016/j.rse.2004.06.014  
**Description:** This raster layer represents built land cover as derived from a combination of Landsat Thematic Mapper ™ imagery and Japanese Earth Resources Satellite-1 (JERS-1) synthetic aperture radar (SAR) data.  
**Class:** raster  
**Derived Covariates:**  
prp, cls, dst,

```
class       : RasterLayer 
dimensions  : 11264, 8912, 100384768  (nrow, ncol, ncell)
resolution  : 100, 100  (x, y)
extent      : -66865, 824335, -519218, 607182  (xmin, xmax, ymin, ymax)
coord. ref. : +proj=utm +zone=37 +datum=WGS84 +units=m +no_defs +ellps=WGS84 +towgs84=0,0,0 
data source : D:\Documents\Graduate School\Research\Population\Data\RF\data\KEN\Built\Derived\built_cls.tif 
names       : built_cls 
values      : 0, 1  (min, max)
attributes  :
 ID OID Value     Count
  0   0     0 134192546
  1   1     1    192862
```

---

### Kenyan Health Clinics

**Folder:** Clinics  
**File Name:** noor\_clinics\_type3.shp  
**Source:** Noor, A. M., Alegana, V. A., Gething, P. W., & Snow, R. W. (2009). A spatial national health facility database for public health sector planning in Kenya in 2008. International Journal of Health Geographics, 8, 13. doi:10.1186/1476-072X-8-13  
**Description:** These data were derived from those acquired by and described by Noor, et al. (2009). They represent point locations for major health-related points of interest within the Kenyan national boundary. Clinics, dispensaries and hospitals were separated into individual datasets from the original shapefile provided by Noor, et al.  
**Class:** point  
**Derived Covariates:**  
prp, cls, dst,

```
class       : SpatialPointsDataFrame 
nfeatures   : 934 
extent      : 34, 42, -4.7, 5.4  (xmin, xmax, ymin, ymax)
coord. ref. : NA 
nvariables  : 9
```

---

### Kenyan Health Dispensaries

**Folder:** HDispensaries  
**File Name:** noor\_dispensaries\_type4-6.shp  
**Source:** Noor, A. M., Alegana, V. A., Gething, P. W., & Snow, R. W. (2009). A spatial national health facility database for public health sector planning in Kenya in 2008. International Journal of Health Geographics, 8, 13. doi:10.1186/1476-072X-8-13  
**Description:** These data were derived from those acquired by and described by Noor, et al. (2009). They represent point locations for major health-related points of interest within the Kenyan national boundary. Clinics, dispensaries and hospitals were separated into individual datasets from the original shapefile provided by Noor, et al.  
**Class:** point  
**Derived Covariates:**  
prp, cls, dst,

```
class       : SpatialPointsDataFrame 
nfeatures   : 3711 
extent      : 34, 42, -4.7, 5.4  (xmin, xmax, ymin, ymax)
coord. ref. : NA 
nvariables  : 9
```

---

### Major Hospitals

**Folder:** Hospitals  
**File Name:** noor\_hospitals\_type1-2.shp  
**Source:** Noor, A. M., Alegana, V. A., Gething, P. W., & Snow, R. W. (2009). A spatial national health facility database for public health sector planning in Kenya in 2008. International Journal of Health Geographics, 8, 13. doi:10.1186/1476-072X-8-13  
**Description:** These data were derived from those acquired by and described by Noor, et al. (2009). They represent point locations for major health-related points of interest within the Kenyan national boundary. Clinics, dispensaries and hospitals were separated into individual datasets from the original shapefile provided by Noor, et al.  
**Class:** point  
**Derived Covariates:**  
prp, cls, dst,

```
class       : SpatialPointsDataFrame 
nfeatures   : 299 
extent      : 34, 42, -4.5, 4.3  (xmin, xmax, ymin, ymax)
coord. ref. : NA 
nvariables  : 9
```

---

### School Locations

**Folder:** Schools  
**File Name:** KEN\_School.shp  
**Source:** Kenya Open Data. (2013). Open Kenya, Transparent Africa. Kenya Primary Schools, 2007. Retrieved May 24, 2013, from https://opendata.go.ke/Education/Kenya-Primary-Schools-2007/p452-xb7c  
**Description:** These data represent school and education-related points of interest and were provided by the Kenya Open Data Initiative ( https://opendata.go.ke ).  
**Class:** point  
**Derived Covariates:**  
prp, cls, dst,

```
class       : SpatialPointsDataFrame 
nfeatures   : 3759 
extent      : -60144, 817711, -454529, 473877  (xmin, xmax, ymin, ymax)
coord. ref. : NA 
nvariables  : 5
```

---
