# Supplementary figures and images for: Disaggregating Census Data for Population Mapping Using Random Forests with Remotely-Sensed and Ancillary Data
Source: PLoS One. 2015 Feb 17;10(2):e0107042. doi: 10.1371/journal.pone.0107042 (PMC4331277; doi:10.1371/journal.pone.0107042)

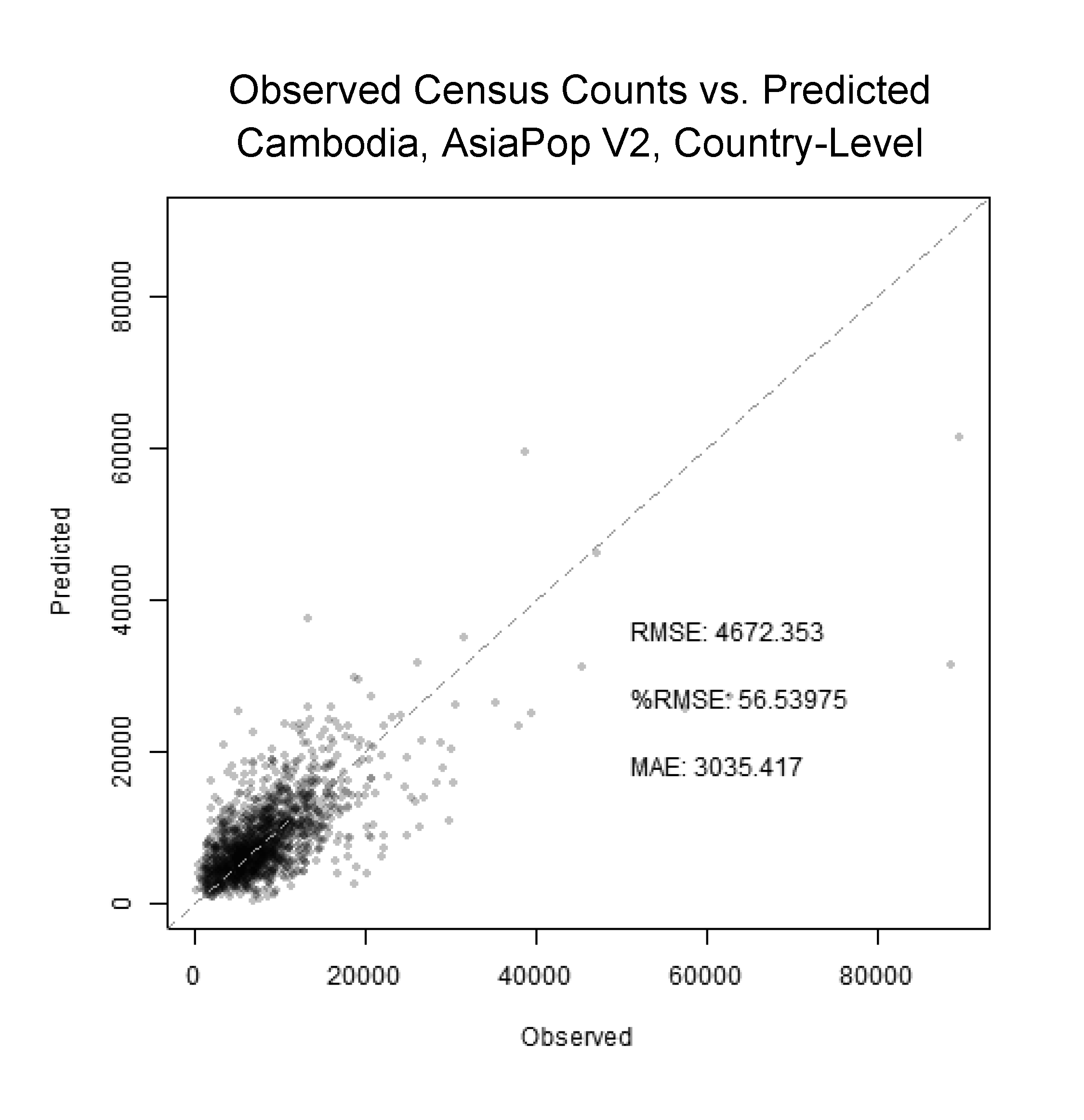

Supplement: S3 File — We illustrate the utility of the Random Forest methodology and the prediction density layer it produces by summing all census units into a single country-wide population count. We then distribute the total population number according to the relative weight for each pixel in our prediction layer, just as we would for individual census units. Doing this for Cambodia (S3 File) and comparing the distribution to results where individual census unit counts were used as the units for dasymetric redistribution (Fig. 4a) illustrates the utility of the prediction layer as a whole. The results show that though we lose some predictive power (illustrated by the greater spread around the 1:1 line) we are not increasing bias in our estimates (illustrated by the linear trend of the observed vs. predicted values along the 1:1 line). The resulting RMSE of 4672 indicates that even when eliminating the “anchor” effect of distributing census unit counts, the Random Forest methodology still outperformed the approaches used by GRUMP and GPW methods, even though they are both using finer-level census data to anchor their predictions. (TIF) [file pone.0107042.s003.tif]
